# Relationship between menstruation-related experiences and health-related quality of life of Japanese high school students: a cross-sectional study

**DOI:** 10.1186/s12905-023-02777-3

**Published:** 2023-11-21

**Authors:** Motoyuki Nakao, Yuko Ishibashi, Yumika Hino, Keiko Yamauchi, Kotaro Kuwaki

**Affiliations:** Department of Public Health, School of Medicine, Kurume University, 67 Asahimachi, Kurume-shi, Fukuoka, 830-0011 Fukuoka Japan

**Keywords:** Menstruation, Quality of life, Adolescent

## Abstract

**Background:**

Recently, there has been a growing global movement concerning menstruation, a healthy and natural physiological phenomenon in women. The disadvantages caused by menstruation are “gender-based obstacles.“ Adolescent girls are also under its influence and perhaps in a more vulnerable situation than adult women. This study investigated the experiences related to menstruation that affect health-related quality of life (HRQOL) of high school students in Japan.

**Methods:**

This cross-sectional study was conducted at a municipal high school in Fukuoka Prefecture, Japan. The study population comprised 233 female students among which 198 completed the questionnaire. The questionnaire contained items about menstruation and HRQOL measured by the 36-Item Short Form Health Survey (SF-36).

**Results:**

Approximately a quarter had experienced difficulties in obtaining sanitary products in the past year, whether for economic or non-economic reasons. Menstruation-associated symptoms, impact on daily life, trouble with menstruation at an unexpected time, usage of painkillers, unhealthy lifestyle, and negative perception of menstruation were significantly associated with lower HRQOL scores, particularly in the mental component summary scores of the SF-36.

**Conclusions:**

For the high school students with severe menstruation-associated symptoms that interfere with their daily lives, the results of this study suggest that improving access to medical care, information, and education can contribute to a better HRQOL.

**Supplementary Information:**

The online version contains supplementary material available at 10.1186/s12905-023-02777-3.

## Background

Over the last decade, there has been a growing global trend concerning menstruation [1, 2]. What was once discussed from a clinical or individual perspective, such as reproductive health, disorders, and family planning, has since been focused on as a public health issue; it could be an appeal to repeal the taxation on sanitary products in high-income countries or issues of clean water, sanitation, hygiene, and human rights due to poverty, tradition, and taboos in low- and middle-income countries [3, 4]. Especially in more recent times, menstrual issues are often discussed in the context of “period poverty” which refers to the inadequate access to a hygienic environment for menstruation, including sanitary facilities and menstrual products, as well as education about menstruation [5]. Although this topic is often discussed primarily as a hygiene issue in low- and middle-income countries, women in high-income countries also face problems of not being able to afford sanitary products [4, 6]. Due to gender pay gap and high rates of non-regular employment, women are more vulnerable to poverty in the event of a socioeconomic shock such as the COVID-19 pandemic [7, 8]. Japan is one of the wealthiest countries in the world; however, some people here suffer from “period poverty” [9, 10]. In an online survey by a Japanese student group, approximately 20% of respondents reported that they had difficulty purchasing sanitary products for financial reasons [11]. In Japan, the issue of “period poverty” became widely known due to the rapid increase in the number of women living in poverty as a result of the stagnation of socioeconomic activities due to the COVID-19 pandemic [12]. Therefore, “period poverty” is often perceived as a temporary problem caused by COVID-19 in Japan. In fact, the government of Japan has put forth support for “period poverty” as part of its “COVID-19 countermeasures” [13]. However, as mentioned above, “period poverty” is a concept that encompasses a much broader meaning and is an ongoing issue [5]. Regardless of socioeconomic status, most women experience menstruation monthly for decades. Furthermore, menstruation, although a normal and healthy bodily function, can cause pain, discomfort, and for some people, anxiety, shame, and embarrassment [14]. Some adolescents are affected by these experiences which lead to school absenteeism [15]. In Japan, although working women can take menstrual leave, students do not have such option, and a high absentee rate leads to poor academic evaluation, which is detrimental to higher education and employment [16]. Considering the above, the disadvantages caused by menstruation, which occurs only in women, are evidently “gender-based obstacles”; adolescent girls are also under its influence and perhaps are in a more vulnerable situation than adult women. Since most Japanese adolescents, including high school students, are supported by their parents, it is likely that their parents purchase sanitary products and other daily necessities for them. However, as is evident from the fact that " period poverty” encompasses a broad concept, the disadvantages caused by menstruation do not disappear if only sanitary products are available. The online survey mentioned above also reports that high school students suffer various disadvantages due to taboo, prejudice, stigma, and lack of information about menstruation [11]. Even though we know that various problems related to menstruation exist in adolescent women, no studies have examined the relationship between such disadvantages and health status. This is an issue that should be clarified for the health and well-being of adolescent females. Since health problems related to menstruation may not be clinical for adolescent women, we decided to use health-related quality of life (HRQOL) as a measure of health problems. This study is the first to examine the relationship between various experiences, subjective symptoms, and perceptions related to menstruation and HRQOL among female high school students in Japan.

## Methods

### Study design and participants

This cross-sectional study was conducted on March 1, 2022, at a municipal high school located in Fukuoka Prefecture, Japan. Female high school students who belonged to the school where the survey was conducted were eligible for the study (n = 233); those who were present on the day of the survey were included in the study (n = 198), and those who were absent on the day of the survey were excluded (n = 35). No students declined to participate in the study. The students who were present on the day of the survey completed the questionnaire (Table 1).

### Questionnaire

The questionnaire consisted of items on age, menstrual cycle (periodic or irregular), age at menarche, types of sanitary products, use of oral contraceptives (prescribed drugs), use of painkillers (over-the-counter (OTC) drugs), experience of problems with menstruation (accessing menstrual products, limitation of daily activities, starting at unexpected times), subjective lifestyle habits, menstruation-associated symptoms, perceptions about menstruation, and the Short Form-36 Health Survey version 2 (SF-36). Permission to use the SF-36 Japanese version was obtained from Qualitest Inc. (Kyoto, Japan). The SF-36 has been established as a valid and reliable questionnaire for measuring subject-reported outcomes regarding HRQOL [17, 18]. It comprises 36 questions on 3-, 5-, or 6-point ordinal scales from which 8 subscales (physical functioning, role limitations due to physical health problems, bodily pain, general health perceptions, vitality, social functioning, role limitations due to emotional problems, and mental health) from 0 to 100 points (minimum = 0, maximum = 100) were calculated according to the online scoring manual [19]. These subscale scores were converted to a mean of 50 points and standard deviation of 10 points based on the 2017 Japanese national norm using a web-based scoring system (https://qolsys.jp/). Norm-based scoring (NBS) scores were further categorized into three components: physical component summary (PCS), mental component summary (MCS), and role-social component summary (RCS). Component summary scores were calculated based on the 2017 Japanese national norm and factor loadings from the 2002 national survey in Japan. The validity of the model for calculating three-component summary was confirmed by Suzukamo et al. [20]. The mean subscale and NBS scores are presented in Supplementary Table 1.

### Statistical analyses

Data were anonymized and electronically managed for analysis. Statistical analyses, including Wilcoxon rank sum test and χ^2^ test, were performed using IBM SPSS Statistics (version 25.0; IBM Corporation, Armonk, NY, USA), and p-values < 0.05 were considered to indicate statistical significance.

## Results

The participants’ characteristics are shown in Table 1. The number of female students enrolled in the high school was 233. Of these, 198 (85.0%) were present on the day of the survey and completed the questionnaire. Among the students, 87 (43.9%) reported periodic menstruation. The mean age at menarche was 12.3 ± 1.4 years. Most students (95.3%) used disposable sanitary napkins, whereas less than 10% used tampons or absorbent shorts. Only a few students (1.6%) took oral contraceptives, while more than half of the students (57.6%) took painkillers during their menstruation. A quarter of the students had experienced difficulties obtaining sanitary products in the past year for economic or non-economic reasons. The most prominent impact of menstruation on daily life was “absenteeism, tardiness, or leaving early from school”, which 41.9% of the students had experienced at least once within the past year. Following this, students experienced “avoidance of physical activity” (38.7%), and “absenteeism form part-time job” (18.0%). More than half (63.4%) of the students indicated that they had experienced trouble with menstruation starting at an unexpected time. More than two-thirds of the students reported having a well-balanced diet (67.5%) and sufficient sleep (67.2%). Students who exercised routinely accounted for 31.3% and 22.0% were on a restricted diet for weight loss. The mean number of subject-reported menstruation-associated symptoms was 7.4 with a standard deviation of 4.1. Each symptom is shown in Supplementary Table 2. The most common menstruation-associated symptom was body (abdominal or back) pain (84.1%). Participants’ perceptions of menstruation were divided into positive and negative aspects. The most common positive and negative perceptions were “important” (28.3%) and “bothersome” (69.7), respectively.

**Table 1:** Participant characteristics

	n
**Total number of students**	233
	n (% of total)
**Students present on the day of survey**	198 (85.0)
**School year**	n (%)
First year	66 (33.3)
Second year	76 (38.3)
Third year	56 (28.3)
**Periodic menstrual cycle** ^**1**^	87 (43.9)
	(Mean ± SD)
**Age at menarche**	12.3 ± 1.4
**Regularly used sanitary products** ^**2**^	n (%)
Pads	183 (95.3)
Tampon	15 (7.8)
Absorbent shorts	3 (1.6)
**Taking oral contraceptives**	3 (1.6)
**Taking pain killer at menstruation**	110 (57.6)
**Experience of trouble accessing menstrual products**	
Economic reasons^3^	46 (23.2)
Non-economic reasons^4^	48 (24.2)
**Experience of limitation of activities**	
Absenteeism, tardiness, or leave early from school	80 (41.9)
Absence from examinations	10 (5.3)
Non-participation in social activities	15 (7.9)
Avoiding activities including physical exercise, such as physical education and sports	74 (38.7)
Absenteeism from part-time job	34 (18.0)
**Experience of trouble with menstruation at an unexpected time**	121 (63.4)
**Subjective lifestyles**	
Having a well-balanced diet	129 (67.5)
Having sufficient sleep	129 (67.2)
Restricted diet for weight loss	42 (22.0)
Exercise routinely	60 (31.3)
	(Mean ± SD)
**Numbers of menstruation-related symptoms**	7.4 ± 4.1
**Perceptions about menstruation**	
** Positive perceptions**	n (%)
Important	56 (28.3)
Nothing special	18 (9.1)
Pleased	6 (3.0)
Proud	5 (2.5)
** Negative perceptions**	
Bothersome	138 (69.7)
Debilitating	119 (60.1)
Embarrassed	15 (7.6)
Dirty	8 (4.0)

1: Participants who answered “I have periodic menstruation” to the question about the menstrual cycle

2: Multiple answers were permitted to this query. 3: Economic reasons include “unable, struggle, or hesitate to purchase sanitary products due to economic reasons”; “ask sanitary pads from others due to economic reasons”; “reduced frequency of changing pads due to economic reasons”; and “used alternatives such as toilet paper instead of sanitary pads due to economic reasons.” 4: Non-economic reasons include “embarrassed to buy sanitary products”; “embarrassed to ask parents to buy sanitary products”; “suddenly started menstruating but did not have sanitary products and could not go to purchase them”; “forgot to bring pads to school on a menstruating day”

**Fig. 1:**
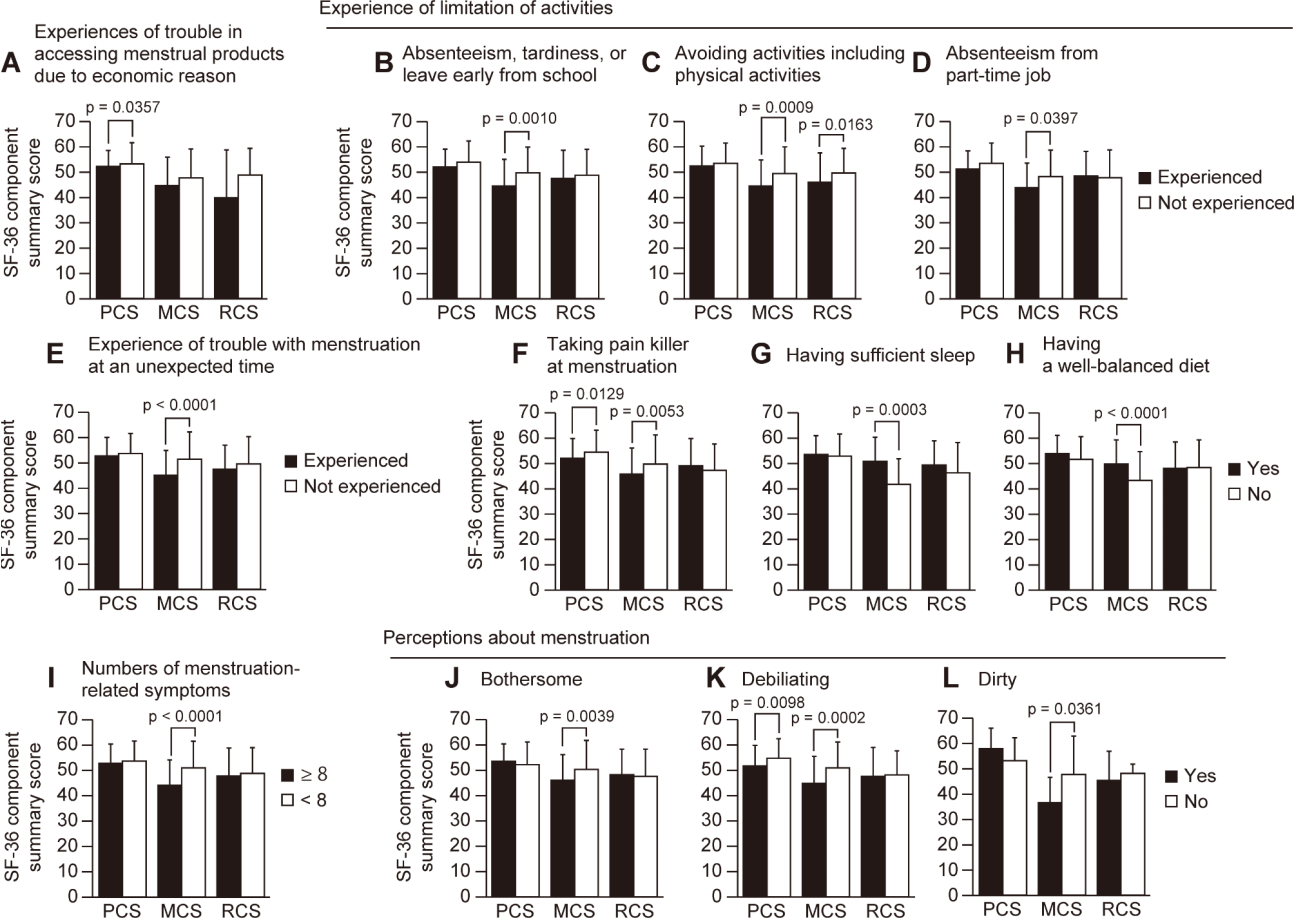
Relationships between the menstruation-related experiences, lifestyle habit, symptom, and perceptions toward menstruation and HRQOL. Data are presented as mean ± SD. The p-values as results of the Wilcoxon rank sum tests are shown at the top of the column with significant differences. There were no significant differences in columns with no p-values indicated. PCS, physical component summary; MCS, mental component summary; RCS, role-social component summary

The SF-36 component summary scores were compared between different menstrual-associated experiences, symptoms, and lifestyle habits, and perceptions (Fig. 1). The students experienced trouble in accessing menstrual products due to economic reason showed significant lower PCS score than that of those not experienced, although the score was above national norm (Fig. 1A). Students with menstruation-related experiences of absenteeism, tardiness, and leaving early from school; avoidance of activities including physical exercise such as physical education (PE) and sports; absenteeism from a part-time job; and trouble with menstruation starting at an unexpected time showed significantly lower scores on MCS (Fig. 1B – E). Students taking painkillers during menstruation showed significantly lower PCS and MCS scores (Fig. 1F). Regarding lifestyle, students with sufficient sleep and a well-balanced diet showed significantly higher MCS scores (Fig. 1G and H). The students who reported eight (median) or more menstruation-associated symptoms and felt bothersome and/or dirty toward menstruation showed significantly lower MCS scores (Fig. 1I, J and L). Significantly lower PCS and MCS scores were observed among students who felt debilitating about menstruation (Fig. 1K). The association between menstruation-related experiences and SF-36 RCS score was only observed among students who avoided physical exercise during menstruation (Fig. 1C). The data regarding other characteristics that did not show significant differences in the SF-36 component summary scores are shown in Supplementary Table 3.

## Discussion

This study examined the relationship between menstruation-related experiences and characteristics and HRQOL among high school students in Japan.

The results of the present study showed that there were students who had difficulty obtaining sanitary products for non-economic as well as economic reasons. Students who had experienced difficulties in obtaining sanitary products due to financial reasons had significantly lower PCS scores than those who had never experienced such difficulties (Fig. 1A). Cardoso et al. reported that the experience of “period poverty” was associated with depressive symptoms among university students in the United States [21]. Whereas, in the present study, the experience of difficulty in obtaining sanitary products due to economic reasons did not show a significant association with MCS. Compared to university students, many of whom live away from their parents, most high school students depend on their parents for their living arrangements, including finance. Therefore, there may be a difference in the seriousness of difficulty in purchasing sanitary products. The differences between the household economic situations of high school and university students should be further examined in detail. On the other hand, experiences related to menstruation (Fig. 1B – E) had a marked impact on the HRQOL of high school students, especially on MCS scores. Additionally, MCS scores were significantly lower in students with more menstruation-associated symptoms (Fig. 1I). It may be assumed that these menstruation-associated symptoms are responsible for limiting activities of daily life. In fact, this association was confirmed by a comparison of limitation of activities and the number of menstruation-associated symptoms, which showed that those with activity limitation had a significantly higher number of symptoms (Supplementary Table 4). In this study, the severity of the symptoms was not examined; thus, it is unclear whether the symptoms were pathological conditions. However, the finding that menstruation-associated symptoms affect HRQOL is consistent with reports that show HRQOL is lower among adolescents with dysmenorrhea and other menstruation-related problems [21–24]. Most of the students had symptoms associated with menstruation, with 88 (55.6%) taking OTC medications during menstruation, while only three students visited a clinic to receive prescriptions for medication (e.g., low-dose estrogen/progestin). In addition, five students wanted to consult a medical professional for menstruation-related problems, but were unable to do so due to embarrassment or their parents’ belief that it is “immoral” for high school students to use contraceptive drugs (data not shown). The possibility cannot be ignored that taboos/stigmas associated with menstruation, such as being “ashamed” to seek medical care or medical advice because of menstruation-related issues and being “immoral” in the eyes of their parents, other adults, friends, or boys, may have an impact on adolescents’ mental and physical health. It has been reported that adolescent girls are less likely to see a doctor if they have problems related to menstruation [25, 26]. Since Japan has universal health coverage and, in principle, all citizens are covered by public medical insurance, access to medical care is easy and the cost should not be a barrier if the individual feels the need for medical care [27]. Furthermore, Fukuoka Prefecture, where this study was conducted, has more physicians per population than the national average and is well resourced for healthcare [28]. However, one reason why few women seek medical care in spite of experiencing pain or other symptoms during menstruation may be that there is a stereotype among women that menstruation is painful and natural, and both women and their parents think that a certain extent of pain is normal and should be accepted [29, 30]. Henry et al. reported that lack of health literacy, menstrual taboos/normalization, and problems with health care providers were the reasons why few people see a doctor even if they have abnormal uterine bleeding [31]. Regarding health literacy, in addition to the stereotypes mentioned above, the participants in this study may lack knowledge about the relationship between dysmenorrhea and other serious diseases, such as endometriosis and uterine fibroids, and may not have the option of “visiting a doctor”. This is also a form of “period poverty” in that there is a lack of access to proper education, knowledge, and information about menstruation.

Regarding lifestyle, the MCS scores were significantly lower among those who did not get enough sleep and did not have a well-balanced diet (Fig. 1G and H). Sleep and diet have been reported to be associated with irregular menstrual cycles and dysmenorrhea [24, 31–34]. Therefore, the fact that MCS scores were lower among those who were aware that they did not have a good lifestyle suggests that education on improving lifestyle habits may lead to improvement in mental health status.

In this study, the MCS score was affected for students who had “bothersome” and “dirty” perceptions about menstruation, and PCS and MCS scores were affected for those who had “debilitating” feelings about menstruation (Fig. 1J – L). It can be said that negative perceptions about menstruation are associated with symptoms related to menstruation and various unpleasant experiences related to it. Indeed, negative perceptions toward menstruation have been reported to be associated with symptoms associated with menstruation and knowledge of the menstrual cycle [35]. Additionally, research on university students has reported that negative perceptions about menstruation, along with social stigma, are associated with lower confidence in managing menstruation [36]. In the present study, the experience of trouble with menstruation starting at an unexpected time was significantly associated with lower MCS scores (Fig. 1E). The trouble with menstruation at an unexpected time would most likely be experienced in managing menstruation at school; for example, not carrying sanitary products or not being able to change them. In the present study, negative perceptions of menstruation were significantly correlated with the experience of trouble with menstruation at an unexpected time, suggesting that the experience of trouble with menstruation at school may have led to negative perceptions of menstruation (Supplementary Table 5). To alleviate the impact of problems caused by unexpected menstruation, the provision of free sanitary products in school restrooms and other facilities may be effective. In general, the provision of free sanitary products is often thought to be for those who are economically deprived; however, it is also necessary to protect the dignity of everyone, including those who have irregular menstrual cycles, regardless of their economic status.

This study had several limitations. First, because of the cross-sectional design of this study, it was not possible to verify the causality between the factors of menstruation and HRQOL. Second, this study was conducted on a single day; therefore, students who were absent on that day were not included. The reasons for the absence of these students were unknown; however, they might have more serious problems with menstruation. In such cases, the possibility of a selection bias cannot be excluded. Additionally, since this survey was conducted in only one high school, it is possible that the sample may not be representative of the general high school population. Since this is a pilot study, further surveys should be conducted in a larger number of high schools to address generalizability. Finally, multivariate analysis was not performed in this study because of the small sample size. Therefore, the variables that were significantly different in this study might be due to confounding factors. It is necessary to conduct a study with a larger sample size and perform a multivariate analysis to verify the confounding factors.

## Conclusions

This study investigated the relationship between menstruation-related experiences, symptoms, and perceptions with HRQOL as measured by the SF-36 in female high school students. The impact of menstruation, which is a healthy and natural physiological phenomenon for women, on daily life, more menstruation-associated symptoms, negative perception of menstruation, unhealthy lifestyle, and the use of painkillers were significantly associated with lower scores on the psychological aspects of HRQOL. For those with severe symptoms that interfere with their daily lives, the study suggests that improving access to medical care, information, and education can contribute to better mental health.

### Electronic supplementary material

Below is the link to the electronic supplementary material.

Supplementary Material 1

## Data Availability

The datasets analyzed during the current study are not publicly available because of our privacy policy and agreement with participants, but are available from the corresponding author upon reasonable request.

## Abbreviations

HRQOL: Health-related quality of life
SF-36: 36-Item Short Form Health Survey
OTC: Over the counter
PCS: Physical component summary
MCS: Mental component summary
RCS: Role-social component summary
SD: Standard deviation
