# Supplementary material for: Relationship between menstruation-related experiences and health-related quality of life of Japanese high school students: a cross-sectional study
Source: BMC Womens Health. 2023 Nov 21;23:620. doi: 10.1186/s12905-023-02777-3 (PMC10664610; doi:10.1186/s12905-023-02777-3)
Supplement: Supplementary file 1 — Supplementary Material 1 [file 12905_2023_2777_MOESM1_ESM.pdf]

Supplementary Table 1.

Scores of the SF-36 version 2 Japanese version and differences in SF-36 component summary scores between various characteristics of participants

**Subscale**	Score	Norm-based scoring[1]
Physical functioning	93.0 ± 11.6	53.3 ± 6.3
Role limitation due to physical problems	87.1 ± 19.9	50.8 ± 9.2
Bodily pain	65.5 ± 26.3	45.4 ± 12.0
General health perceptions	62.9 ± 19.6	52.4 ± 10.1
Vitality	51.9 ± 22.2	47.7 ± 10.8
Social functioning	80.4 ± 23.1	48.9 ± 10.4
Role limitation due to emotional problems	80.4 ± 23.7	48.0 ± 10.6
Mental health	61.7 ± 19.8	47.8 ± 10.0

Data are presented as the mean ± SD. 1: Norm-based scoring (NBS) was performed using the Japanese national norm of 2017.

Supplementary Table 2. Prevalence of menstruation-related symptoms

	n (%)
Pain (abdominal, back)	164 (84.1)
Unmotivated	131 (67.2)
Irritability	123 (63.1)
Drowsiness	122 (62.6)
Fatiguability	120 (61.5)
Short temper	103 (52.8)
Rough skin	99 (50.8)
Depressed	94 (48.2)
Difficulty concentrating	86 (44.1)
Bloating sensation	84 (43.1)
Stiffness in the shoulder and neck	61 (31.3)
Decreased efficiency	59 (30.3)
Get distracted	56 (28.7)
Restlessness	50 (25.6)
Anorexia	37 (19.0)
Enhanced appetite	13 (6.7)
Weight gain	11 (5.6)
Body swelling	9 (4.6)
Miscellaneous[1]	44 (22.6)

1: Symptoms of < 10 respondents were summarized as "Miscellaneous", which included the symptoms of headache, nausea, swelling of breasts, diarrhea, difficulty moving, negative feelings, sensitivity to cold, dizziness, physical weariness, joint pain, and anemia.

Supplementary Table 3

Variable	Group	n	SF-36 component summary[1]		
			Physical	Mental	Role-social
All participants	-	193	53.1 ± 7.9	47.5 ± 10.7	48.2 ± 10.6
Grades	1	65	52.8 ± 8.7	46.7 ± 10.9	46.4 ± 8.9
	2	72	53.9 ± 7.2	46.8 ± 11.1	48.3 ± 10.7
	3	56	52.3 ± 7.8	49.3 ± 9.7	50.3 ± 11.9
	Kruskal-Wallis test		N.S.	N.S.	0.0122
Menstrual cycle	Periodic	84	53.6 ± 6.7	48.3 ± 11.6	49.0 ± 10.8
	Irregular	109	52.7 ± 8.7	46.8 ± 9.9	47.5 ± 10.4
	Wilcoxon rank sum test		N.S.	N.S.	N.S.
Experience of trouble in accessing menstrual products					
Economic reason	Yes	45	51.4 ± 6.9	45.6 ± 10.5	48.2 ± 10.2
	No	148	53.44 ± 8.1	48.1 ± 10.7	48.2 ± 10.7
	Wilcoxon rank sum test		p = 0.0357	N.S.	N.S.
Non-economic reason	Yes	46	53.2 ± 7.0	46.9 ± 9.4	47.3 ± 9.5
	No	147	53.0 ± 8.2	47.7 ± 11.1	48.5 ± 10.9
	Wilcoxon rank sum test		N.S.	N.S.	N.S.
Experience of limitation of activities					
Absenteeism, tardiness, or leave early from school	Yes	78	52.0 ± 7.0	44.4 ± 10.6	47.4 ± 11.1
	No	115	53.8 ± 8.4	49.6 ± 10.2	48.7 ± 10.2
	Wilcoxon rank sum test		N.S.	p = 0.0010	N.S.
Absence from examinations	Yes	10	52.2 ± 10.0	44.6 ± 8.1	39.7 ± 17.7
	No	183	53.1 ± 7.8	47.6 ± 10.8	48.7 ± 9.9
	Wilcoxon rank sum test		N.S.	N.S.	N.S.
Non-participation in social activities	Yes	15	51.8 ± 9.0	43.0 ± 9.2	45.5 ± 14.4
	No	178	53.2 ± 7.8	47.9 ± 10.7	48.4 ± 10.2
	Wilcoxon rank sum test		N.S.	N.S.	N.S.
Avoiding activities including physical exercise, such as physical education and sports	Yes	73	52.4 ± 7.8	44.4 ± 10.3	45.9 ± 11.6
	No	120	53.4 ± 8.0	49.4 ± 10.5	49.6 ± 9.7
	Wilcoxon rank sum test		N.S.	p = 0.0009	p = 0.0163
Absenteeism from part-time job	Yes	33	51.6 ± 7.3	44.0 ± 9.8	48.7 ± 9.5
	No	160	53.4 ± 8.0	48.2 ± 10.7	48.1 ± 10.8
	Wilcoxon rank sum test		N.S.	p = 0.0397	N.S.
Experience of trouble with menstruation at an unexpected time	Yes	119	52.7 ± 7.8	45.0 ± 10.3	47.4 ± 10.8
	No	74	53.6 ± 8.0	51.4 ± 10.2	49.5 ± 10.1
	Wilcoxon rank sum test		N.S.	p < 0.0001	N.S.
Taking pain killer at menstruation	Yes	107	52.0 ± 7.0	45.7 ± 9.7	49.0 ± 10.7
	No	86	54.4 ± 8.7	49.7 ± 11.5	47.2 ± 10.4
	Wilcoxon rank sum test		p = 0.0129	p = 0.0053	N.S.
Having sufficient sleep	Yes	125	53.5 ± 7.4	50.7 ± 9.6	49.3 ± 9.6
	No	68	52.8 ± 8.8	41.7 ± 10.1	46.2 ± 12.0
	Wilcoxon rank sum test		N.S.	p = 0.0003	N.S.
Having a well-balanced diet	Yes	126	53.9 ± 7.2	49.7 ± 9.6	48.1 ± 10.4
	No	67	51.6 ± 9.0	43.3 ± 11.4	48.4 ± 10.9
	Wilcoxon rank sum test		N.S.	p < 0.0001	N.S.
Restricted diet for weight loss	Yes	41	51.7 ± 6.8	45.4 ± 10.9	49.4 ± 8.5
	No	152	53.4 ± 8.1	48.1 ± 10.6	47.9 ± 11.1

	Wilcoxon rank sum test		N.S.	N.S.	N.S.
Exercise routinely	Yes	60	53.2 ± 7.1	48.8 ± 12.7	48.4 ± 9.3
	No	133	53.0 ± 8.2	46.9 ± 9.6	48.1 ± 11.1
	Wilcoxon rank sum test		N.S.	N.S.	N.S.
Number of menstruation-related symptoms	≥ 8	98	52.8 ± 7.6	44.1 ± 10.0	47.8 ± 11.0
	<8	92	53.7 ± 7.9	51.0 ± 10.5	48.8 ± 10.2
	Wilcoxon rank sum test		N.S.	p < 0.0001	N.S.
Perceptions about menstruation					
Positive perceptions					
Important	Yes	56	53.1 ± 8.3	48.9 ± 10.4	48.3 ± 11.4
	No	137	53.1 ± 6.8	46.9 ± 10.8	48.2 ± 10.3
	Wilcoxon rank sum test		N.S.	N.S.	N.S.
Nothing special	No	17	54.3 ± 7.5	51.0 ± 10.2	47.0 ± 10.8
	Yes	176	52.9 ± 7.9	47.2 ± 10.7	48.3 ± 10.6
	Wilcoxon rank sum test		N.S.	N.S.	N.S.
Pleased	Yes	6	50.9 ± 5.6	43.5 ± 5.5	45.0 ± 3.4
	No	187	53.1 ± 7.9	47.6 ± 10.8	48.3 ± 10.7
	Wilcoxon rank sum test		N.S.	N.S.	N.S.
Proud	Yes	5	50.6 ± 7.1	46.9 ± 7.6	47.9 ± 8.4
	No	188	53.1 ± 7.1	47.5 ± 10.8	48.2 ± 10.6
	Wilcoxon rank sum test		N.S.	N.S.	N.S.
Negative perceptions					
Bothersome	Yes	134	53.3 ± 7.5	46.1 ± 10.3	48.3 ± 10.6
	No	59	52.5 ± 8.7	50.7 ± 10.9	48.0 ± 10.5
	Wilcoxon rank sum test		N.S.	p = 0.00	N.S.
Debilitating	Yes	116	51.9 ± 8.0	45.1 ± 10.2	48.0 ± 11.2
	No	77	54.8 ± 7.5	51.0 ± 10.5	48.5 ± 9.7
	Wilcoxon rank sum test		p = 0.0098	p = 0.0002	N.S.
Embarrassed	Yes	15	47.8 ± 12.2	52.1 ± 9.7	46.7 ± 12.5
	No	178	53.5 ± 7.3	47.1 ± 10.7	48.3 ± 10.4
	Wilcoxon rank sum test		N.S.	N.S.	N.S.
Dirty	Yes	7	57.9 ± 9.6	37.0 ± 14.7	46.0 ± 3.7
	No	186	52.9 ± 7.8	47.9 ± 10.3	48.3 ± 10.7
	Wilcoxon rank sum test		N.S.	p = 0.0361	N.S.

Data are presented as the mean ± SD. 1: The component summary was calculated using the Japanese norm of 2017 and factor loadings from the national survey of 2002 in Japan.

Supplementary Table 4.
Association of the number of menstruation-related symptoms with the experience of limitation of activities.

	Number of menstruation-related symptoms		Welch's t-test
	Experienced	Not experienced	p-value
Experience of limitation of activities			
Absenteeism, tardiness, or leave early from school	9.4 ± 3.8	6.0 ± 3.6	< 0.0001
Absence from examinations	10.8 ± 5.2	7.2 ± 3.9	0.0550
Non-participation in social activities	10.6 ± 3.7	7.1 ± 4.0	0.0029
Avoiding activities including physical exercise, such as physical education and sports	9.7 ± 4.0	6.0 ± 3.5	< 0.0001
Absenteeism from part-time job	9.4 ± 3.9	6.9 ± 4.0	0.0017
Experience of trouble with menstruation at an unexpected time	8.0 ± 4.2	6.4 ± 3.7	0.0062

Data are presented as the mean ± SD.

Supplementary table 5.
Association between perceptions about menstruation and experience of trouble with menstruation at an unexpected time.

Perceptions about menstruation		Experience of trouble with menstruation at an unexpected time		Fisher’s exact test
		Yes (n = 121)	No (n = 77)	p-value
**Positive perceptions**				
Important	Yes	33	23	0.7470
	No	88	54	
Nothing special	No	7	11	0.0731
	Yes	114	66	
Pleased	Yes	4	2	1.0000
	No	117	75	
Proud	Yes	2	3	0.3790
	No	119	74	
**Negative perceptions**				
Bothersome	Yes	92	46	0.0177
	No	29	31	
Debilitating	Yes	84	35	0.0010
	No	37	42	
Embarrassed	Yes	11	4	0.4132
	No	110	73	
Dirty	Yes	5	3	1.0000
	No	116	74	
